# Autotrophic Fe-Driven Biological Nitrogen Removal Technologies for Sustainable Wastewater Treatment

**DOI:** 10.3389/fmicb.2022.895409

**Published:** 2022-04-29

**Authors:** Suyan Pang, Ning Li, Huan Luo, Xiaonan Luo, Tong Shen, Yanan Yang, Jin Jiang

**Affiliations:** ^1^Key Laboratory of Songliao Aquatic Environment, School of Municipal and Environmental Engineering, Ministry of Education, Jilin Jianzhu University, Changchun, China; ^2^Key Laboratory for City Cluster Environmental Safety and Green Development of the Ministry of Education, School of Ecology, Environment and Resources, Guangdong University of Technology, Guangzhou, China; ^3^Southern Marine Science and Engineering Guangdong Laboratory (Guangzhou), Guangzhou, China; ^4^Guangdong Provincial Engineering Technology Research Center for Life and Health of River & Lake, Pearl River Water Resources Research Institute, Pearl River Water Resources Commission of the Ministry of Water Resources, Guangzhou, China

**Keywords:** biological nitrogen removal, Feammox, nitrate-dependent anaerobic Fe(II) oxidation, iron-reducing microorganisms, iron-oxidizing microorganisms

## Abstract

Fe-driven biological nitrogen removal (FeBNR) has become one of the main technologies in water pollution remediation due to its economy, safety and mild reaction conditions. This paper systematically summarizes abiotic and biotic reactions in the Fe and N cycles, including nitrate/nitrite-dependent anaerobic Fe(II) oxidation (NDAFO) and anaerobic ammonium oxidation coupled with Fe(III) reduction (Feammox). The biodiversity of iron-oxidizing microorganisms for nitrate/nitrite reduction and iron-reducing microorganisms for ammonium oxidation are reviewed. The effects of environmental factors, e.g., pH, redox potential, Fe species, extracellular electron shuttles and natural organic matter, on the FeBNR reaction rate are analyzed. Current application advances in natural and artificial wastewater treatment are introduced with some typical experimental and application cases. Autotrophic FeBNR can treat low-C/N wastewater and greatly benefit the sustainable development of environmentally friendly biotechnologies for advanced nitrogen control.

## Introduction

Nitrogen-containing wastewater has negative effects on human health and aquatic ecosystems. Biological nitrogen removal (BNR) is a low-cost and efficient way to control nitrogen pollution. However, conventional denitrification requires large amounts of carbon-containing compounds, such as methanol, sodium acetate and glucose, causing enormous resource waste, and secondary pollution (Tian and Yu, 2020). Autotrophic denitrification provides multiple alternative methods that adopt inorganic sulfur, hydrogen, and iron as electron donors to reduce nitrate (${\mathrm{NO}}_{3}^{-}$) or nitrite (${\mathrm{NO}}_{2}^{-}$), especially for treating low C/N wastewater (Di Capua et al., 2019). Therefore, the development of autotrophic denitrification techniques has attracted considerable attention in biological wastewater treatment.

Anammox-based techniques have been developed for decades as a representative autotrophic process, but the requisite substrate ${\mathrm{NO}}_{2}^{-}$ heavily relies on short-cut nitrification or denitrification processes (Sheng et al., 2020b). Sulfur-driven autotrophic denitrification utilizes reduced sulfate (S^2−^, S^0^, $S_{2}O_{3}^{2-}$) as an electron donor to reduce ${\mathrm{NO}}_{3}^{-}$, but the acidic environment erodes municipal pipes, leading to billions of dollars in expense per year in Australia (Pikaar et al., 2014). Hydrogen-driven denitrification still has several unaddressed issues, such as safety concerns, high costs, and complex equipment. Although these technologies provide useful solutions to treat low C/N sewage, it is still necessary to explore more efficient and safer autotrophic denitrification technologies.

Fe-driven biological nitrogen removal (FeBNR) consists of two processes, i.e., anaerobic ammonium (${\mathrm{NH}}_{4}^{+}$) oxidation coupled with Fe(III) reduction (Feammox) and nitrate/nitrite-dependent anaerobic Fe(II) oxidation (NDAFO). In the Feammox process, ${\mathrm{NH}}_{4}^{+}$ is oxidized to N_2_, ${\mathrm{NO}}_{2}^{-}$ and ${\mathrm{NO}}_{3}^{-}$ by iron-reducing microorganisms (IRM) in paddy soils, estuary regions, and riparian zones (Ding et al., 2017; Yi et al., 2019). It was estimated that a nitrogen loss of 8.3–17.8 kg-N/(ha·yr) was associated with Feammox in Taihu estuary soils (Ding et al., 2019). In the NDAFO process, ${\mathrm{NO}}_{x}^{-}$ is reduced to N_2_ by iron-oxidizing microorganisms (IOM) using zero-valent iron (ZVI) or Fe(II) as electron donors (Kiskira et al., 2017). Moreover, an Anammox-like process involving the integration of Feammox and NDAFO was used to remove ${\mathrm{NH}}_{4}^{+}$ using ${\mathrm{NO}}_{x}^{-}$ as a terminal electron acceptor through the Fe(III)/Fe(II) cycle (Yang et al., 2020). Since Fe(III)/Fe(II) easily form solid iron compounds in neutral aquatic environments, a single Feammox or NDAFO process is challenging to operate for a long time and easy to produce large amounts of iron sludge (Li et al., 2021b). Ideally, FeBNR can effectively solve the above problem of sludge mineralization and constantly consume ${\mathrm{NH}}_{4}^{+}$ and ${\mathrm{NO}}_{3}^{-}$. The environmentally friendly characteristics, abundance of its constituent species and wide distribution make FeBNR an emerging technique to remediate environmental pollution. The biotic and abiotic reactions between Fe and N are ubiquitous and complex, and a systematic review should be provided for an in-depth understanding of the application of FeBNR to wastewater treatment.

The low reaction rate and the long incubation time of functional microorganisms still hinder FeBNR applications in practical sewage treatment. Unlike conventional heterotrophic denitrification using soluble organic carbon as an electron donor, FeBNR adopts insoluble Fe(III)/Fe(II) minerals to transfer electrons specifically during microbial extracellular respiration. The solubility and bioavailability of solid iron minerals limit the nitrogen removal efficiency. Therefore, the iron metabolizing microorganism and the effect of environmental factors on nitrogen removal efficiency should be summarized. Moreover, various abiotic iron reactions occur extensively in anaerobic environments, increasing the diversity of nitrogen transformation pathways. The underlying mechanisms of nitrogen transformation pathways, including biotic or abiotic processes, should be further identified and discussed.

The purpose of this paper is to review (1) the abiotic and biotic reactions between Fe and N species, elucidating the important effect of the iron cycle on nitrogen transformation; (2) the environmental factors affecting FeBNR efficiency, providing technical references for controlling nitrogen pollution; and (3) the current wastewater treatment processes based on Feammox and NDAFO. On this basis, potential development and application trends of FeBNR technology are proposed.

## Abiotic and Biotic Mechanisms Underlying the Interaction of the Fe and N Cycles

### Abiotic Fe Element Reactions

#### Zero-Valent Iron

zero-valent iron is an effective and abundant reducing agent with a standard redox potential of −0.44 V for ${\mathrm{NO}}_{x}^{-}$ removal from groundwater and wastewater. According to the particle size, ZVI can be classified as nanoscale ZVI (nZVI) and microscale ZVI (mZVI), and nZVI generally has a higher reductive capacity than mZVI due to its greater specific surface area. The mechanisms of the ZVI reaction with ${\mathrm{NO}}_{x}^{-}$ mainly involve (1) direct electron transfer from ZVI to ${\mathrm{NO}}_{3}^{-}$ to form lower-valence nitrogen species, as shown in Eq. 1–3 (Jeong et al., 2012) in Table 1; (2) indirect reduction of ${\mathrm{NO}}_{2}^{-}$ by the produced Fe(II), as in Eq. 4–5 (Liu and Wang, 2019); and (3) removal of ${\mathrm{NO}}_{3}^{-}$ *via* the H_2_ secondarily generated by hydrogenotrophic nitrate-respiring bacteria, as in Eq. 6–8 (Kim and Cha, 2021). In addition, ${\mathrm{NO}}_{3}^{-}$ can be transformed to ${\mathrm{NH}}_{4}^{+}$ by adding both ZVI and Fe(II) with strong reducibility (Eq. 9). Fe(II)EDTA generally has a high adsorption capacity for NO, and the ZVI added in the reactor could convert Fe(II)EDTA-NO to ${\mathrm{NH}}_{4}^{+}$, providing substrates for Anammox and decreasing the toxicity of NO in activated sludge (Eq. 10; Zhang et al., 2019a).

**Table 1:** Abiotic and biotic reactions of ZVI, Fe(II) and Fe(III) with nitrogen species.

Iron species	Reactions	Types	Comments	Eq.	References
ZVI	$4{\mathrm{Fe}}^{0}+2{\mathrm{NO}}_{3}^{-}+10H^{+}\to 4{\mathrm{Fe}}^{2+}+{\mathrm{NH}}_{4}^{+}+3H_{2}O$	Abiotic	ZVI directly reacts with nitrate.	(1)	Jeong et al., 2012
	$5{\mathrm{Fe}}^{0}+2{\mathrm{NO}}_{3}^{-}+12H^{+}\to 5{\mathrm{Fe}}^{2+}+N_{2}+6H_{2}O$	Abiotic		(2)	
	${\mathrm{Fe}}^{0}+2{\mathrm{NO}}_{3}^{-}+4H^{+}\to {\mathrm{Fe}}^{2+}+2{\mathrm{NO}}_{2}^{-}+2H_{2}O$	Abiotic		(3)	
	${\mathrm{Fe}}^{0}\to {\mathrm{Fe}}^{2+}+2e^{-}$	Abiotic	The formed Fe(II) reacts with nitrite.	(4)	Liu and Wang, 2019
	$6{\mathrm{Fe}}^{2+}+{\mathrm{NO}}_{2}^{-}+8H^{+}\to 6{\mathrm{Fe}}^{3+}+{\mathrm{NH}}_{4}^{+}+2H_{2}O$	Abiotic		(5)	
	${\mathrm{Fe}}^{0}+2H_{2}O\to H_{2}+{\mathrm{Fe}}^{2+}+2{\mathrm{OH}}^{-}$	Abiotic	The produced H_2_ reacts with nitrate.	(6)	Kim and Cha, 2021
	${\mathrm{Fe}}^{0}+2H^{+}\to {\mathrm{Fe}}^{2+}+H_{2}$ (acidic conditions)	Abiotic		(7)	
	$5H_{2}+2{\mathrm{NO}}_{3}^{-}\to N_{2}+4H_{2}O+2{\mathrm{OH}}^{-}$	Biotic		(8)	
	$2.82{\mathrm{Fe}}^{0}+0.75{\mathrm{Fe}}^{2+}+{\mathrm{NO}}_{3}^{-}+2.25H_{2}O\to 1.19{\mathrm{Fe}}_{3}O_{4}+{\mathrm{NH}}_{4}^{+}+0.5{\mathrm{OH}}^{-}$	Abiotic	The other transformations of iron species occur.	(9)	Zhang et al., 2019a
	$5{\mathrm{Fe}}^{0}+2{\mathrm{Fe}}^{2+}\mathrm{EDTA}-{\mathrm{NO}}^{2-}+12H^{+}\to 2{\mathrm{Fe}}^{2+}{\mathrm{EDTA}}^{2-}+5{\mathrm{Fe}}^{2+}+2{\mathrm{NH}}_{4}^{+}+2H_{2}O$	Abiotic		(10)	
Fe(II)	${\mathrm{Fe}}^{2+}{\mathrm{EDTA}}^{2-}\left(\mathrm{aq}\right)+\mathrm{NO}\left(\mathrm{aq}\right)↔{\mathrm{Fe}}^{2+}{\mathrm{EDTA}}^{2-}-\mathrm{NO}\left(\mathrm{aq}\right)$	Abiotic	The Fe(II)-EDTA absorption combines Anammox.	(11)	
	$6{\mathrm{Fe}}^{2+}\mathrm{EDTA}-{\mathrm{NO}}^{2-}\left(\mathrm{aq}\right)+4{\mathrm{NH}}_{4}^{+}\to 5N_{2}+6H_{2}O+4H^{+}+6{\mathrm{Fe}}^{2+}{\mathrm{EDTA}}^{2-}\left(\mathrm{aq}\right)$	Biotic		(12)	
	$4{\mathrm{Fe}}^{2+}+2{\mathrm{NO}}_{2}^{-}+9H_{2}O\to 4\mathrm{Fe}{\left(\mathrm{OH}\right)}_{3}+N_{2}O+6H^{+}$	Abiotic	Nitrite chemically reacts with ferrous ion.	(13)	Jones et al., 2015
	$4{\mathrm{Fe}}^{2+}+2{\mathrm{NO}}_{2}^{-}+5H_{2}O\to 4\mathrm{FeOOH}+N_{2}O+6H^{+}$	Abiotic		(14)	
	$4{\mathrm{Fe}}^{2+}+2{\mathrm{HNO}}_{2}+4H^{+}\to 4{\mathrm{Fe}}^{3+}+N_{2}O+3H_{2}O$	Abiotic	Intermediates of nitritation abiotically react with ferrous ion.	(15)	Yang et al., 2018
	$10{\mathrm{Fe}}^{2+}+2{\mathrm{NO}}_{3}^{-}+24H_{2}O\to 10\mathrm{Fe}{\left(\mathrm{OH}\right)}_{3}+N_{2}+18H^{+}$	Biotic	Nitrate reacts with ferrous ion *via* autotrophic denitrifying bacteria.	(16)	Tian et al., 2020
	$10{\mathrm{Fe}}^{2+}+2{\mathrm{NO}}_{3}^{-}+14H_{2}O\to 10\mathrm{FeOOH}+N_{2}+18H^{+}$	Biotic		(17)	
	$15{\mathrm{Fe}}^{2+}+2{\mathrm{NO}}_{3}^{-}+14H_{2}O\to 54{\mathrm{Fe}}_{3}O_{4}+N_{2}+28H^{+}$	Biotic		(18)	
Fe(III)	$4{\mathrm{Fe}}^{3+}+2{\mathrm{NH}}_{2}\mathrm{OH}\to 4{\mathrm{Fe}}^{2+}+N_{2}O+H_{2}O+4H^{+}$	Abiotic	Intermediates of nitritation abiotically react with ferric ion.	(19)	Yang et al., 2018
	$3\mathrm{Fe}{\left(\mathrm{OH}\right)}_{3}+5H^{+}+{\mathrm{NH}}_{4}^{+}\to 3{\mathrm{Fe}}^{2+}+9H_{2}O+0.5N_{2}$	Biotic	Ammonium reacts with ferric ion *via* Feammox.	(20)	Tan et al., 2021
	$6\mathrm{Fe}{\left(\mathrm{OH}\right)}_{3}+10H^{+}+{\mathrm{NH}}_{4}^{+}\to 6{\mathrm{Fe}}^{2+}+16H_{2}O+{\mathrm{NO}}_{2}^{-}$	Biotic		(21)	
	$8\mathrm{Fe}{\left(\mathrm{OH}\right)}_{3}+14H^{+}+{\mathrm{NH}}_{4}^{+}\to 8{\mathrm{Fe}}^{2+}+21H_{2}O+{\mathrm{NO}}_{3}^{-}$	Biotic		(22)	

Although ZVI has been adopted to remediate nitrate-containing wastewater pollution, the shell of the iron oxides formed on the surface of ZVI and the agglomeration of ZVI particles significantly affect the nitrate reduction efficiency. Hence, physical and chemical strategies have been developed to enhance the transformation efficiency and iron corrosion. ZVI composites are prepared to increase the adsorption capacity for ${\mathrm{NO}}_{3}^{-}$ as well as to increase the number of active sites for electron transfer to ${\mathrm{NO}}_{3}^{-}$, mainly through (1) doping ZVI with metal or inorganic components such as Cu, Pt, Al and activated carbon (AC); (2) supporting ZVI composites with matrixes such as calcium alginate and AC; and (3) adding reducing-state additives such as sulfur to ZVI. Meng et al. (2020) encapsulated mZVI and AC into porous calcium alginate to achieve good dispersion of mZVI particles and enhance the iron-carbon galvanic cell effect. An acid mine drainage-based nZVI was synthesized to couple ${\mathrm{NO}}_{3}^{-}$ reduction and norfloxacin oxidation with ultrasound irradiation to overcome passivation (Diao et al., 2019).

#### Fe(II)

In early 1966, Chao and Kroontje (1966) studied the feasibility and intermediates of inorganic ${\mathrm{NO}}_{3}^{-}$ transformation through Fe(II) oxidation. Only under acidic and high-temperature conditions, can Fe(II) be chemically oxidized by ${\mathrm{NO}}_{3}^{-}$. Even Fe(II) can theoretically react with ${\mathrm{NO}}_{3}^{-}$ in a thermodynamically favored reaction at near-neutral pH and environmental temperature, but the kinetic rate is extremely low. Although the redox potential of the Fe(III)/Fe(II) couple (−314–14 mV) is lower than that of all nitrogen couples (${\mathrm{NO}}_{3}^{-}/{\mathrm{NO}}_{2}^{-}$, +430 mV; NO_2_^−^/NO, +350 mV; NO/N_2_O, +1,180 mV; N_2_O/N_2_, +1,350 mV) in denitrification, Fe(II) oxidation coupled with ${\mathrm{NO}}_{3}^{-}$ reduction cannot proceed smoothly without a catalyst. The common catalysts are Cu^2+^, iron (hydr)oxides and even cell surface enzymes. For example, under anoxic conditions in soils and sediments, the dissimilatory reduction of nitrate to ammonia has been observed in the presence of green rust compounds [Fe^II^_4_Fe^III^_2_(OH)_12_SO_4_•yH_2_O; Hansen et al., 1996], representing the occurrence of chemical denitrification or chemodenitrification. The unique structure of green rust may self-catalyze this reaction.

In addition, Fe(II)-EDTA could promote the adoption of aqueous NO and provide a substrate for the production of N_2_ by Anammox bacteria (Eq. 11–12). N_2_O is a natural product primarily derived from biotic denitrification, but chemodenitrification processes also contribute to N_2_O release to the atmosphere. Among them, ${\mathrm{NO}}_{2}^{-}$ can be reduced by Fe(II) to produce N_2_O along with Fe(III; hydr)oxides as byproducts (Eq. 13–14; Jones et al., 2015). In the intermediate step of nitritation, the produced HNO_2_ could be chemically reduced by Fe(II) to release N_2_O, as shown in Eq. 15 (Yang et al., 2018). Nitrite can rapidly be absorbed on goethite at low pH, and surface Fe(II)-goethite complexes show variable reactivity with ${\mathrm{NO}}_{2}^{-}$ to produce N_2_O (Dhakal et al., 2021).

#### Fe(III)

Hydroxylamine (NH_2_OH) is an intermediate in short-cut nitrification and can abiotically react with Fe(III) to produce nitrous oxide (N_2_O), as shown in Eq. 19, and this process is greatly affected by pH. Generally, abiotic reactions play an essential role in the production of total N_2_O at pH values less than 5. To prevent excessive greenhouse gas emissions, short-cut nitrification reactors usually control pH at near-neutral conditions to reduce the release of N_2_O from abiotic reactions (Hikino et al., 2021).

### Microbially Mediated ZVI/Fe(II) Oxidation

Iron and nitrogen cycles are tightly coupled under natural anoxic conditions due to the co-occurrence iron minerals and nitrate in soils, sediments and groundwater. NDAFO is a widely reported biogeochemical process using aqueous or solid-state Fe(II) as electron donors for nitrate/nitrite reduction under circumneutral conditions, as shown in Eq. 16–18 (Tian et al., 2020). ZVI-based autotrophic denitrification utilizes elemental iron substances as electron donors to reduce ${\mathrm{NO}}_{x}^{-}$.

#### Nitrate/Nitrite-Dependent IOM

IOM are widely distributed in the bacterial and archaeal domains (as shown in Table 2). Moreover, IOM are mainly classified into Proteobacteria, Actinobacteria (Zhang et al., 2016a), Firmicutes (Li and Pan, 2019) and Euryarchaeota (Hafenbradl et al., 1996). In 1991, Brons et al. (1991) reported that the nitrate-reducing bacteria *Escherichia coli* reduced ${\mathrm{NO}}_{3}^{-}$ to NO and N_2_O in the presence of Fe(II) and L-lactic acid under anaerobic conditions. In 1996, Straub et al. (1996) enriched and isolated three strains of gram-negative bacteria, which can use Fe(II) as an electron donor for ${\mathrm{NO}}_{3}^{-}$ reduction. Subsequently, NDAFO IOM were found in river and lake sediments, paddy soils, water treatment reactors and constructed wetlands (CWs) and were mainly categorized as *α*-Proteobacteria (Kumaraswamy et al., 2006; Sorokina et al., 2012; Jiang et al., 2015; Park et al., 2018), *β*-Proteobacteria (Anirban and Flynn, 2013; Zhang et al., 2016b, 2019c, 2020a; Liu et al., 2019b; Chen et al., 2020a; Ma et al., 2020; Mai et al., 2021; Xu et al., 2021), *γ*-Proteobacteria (Etique et al., 2014; Li et al., 2015, 2018a; He et al., 2017) and *δ*-Proteobacteria (Su et al., 2020), which play important roles in Fe(II) reduction coupled with ${\mathrm{NO}}_{x}^{-}$ oxidation.

**Table 2:** ZVI/Fe(II)-oxidizing microorganisms for ${\mathrm{NO}}_{2}^{-}/{\mathrm{NO}}_{3}^{-}$ reduction.

Phylum	Species	Sample sites	Electron donors	Electron acceptors	Products	Nutrition type	References
Actinobacteria	*Thermoleophilia* sp.	Cultivated sludge	FeSO_4_·7H_2_O	${\mathrm{NO}}_{3}^{-}$	N_2_	Mixotrophic	Zhang et al., 2016a
Firmicutes	*Clostridium* sp. strain PXL2	Anoxic activated sludge	FeCl_2_·4H_2_O	${\mathrm{NO}}_{3}^{-}$	N_2_	Mixotrophic	Li and Pan, 2019
Euryarchaeota	*Ferroglobus placidus*	Shallow beach	FeS	${\mathrm{NO}}_{3}^{-}$	N_2_	Autotrophic	Hafenbradl et al., 1996
*α*-Proteobacteria	*Hoeflea siderophila* sp.	River sediments	FeS	${\mathrm{NO}}_{3}^{-}$, N_2_O	N_2_	Mixotrophic	Sorokina et al., 2012
	*Paracoccus denitrificans*	Lab-scale bioreactor	FeCl_2_	${\mathrm{NO}}_{3}^{-}/{\mathrm{NO}}_{2}^{-}$	N_2_	Mixotrophic	Park et al., 2018
	*Paracoccus ferrooxidans* BDN-1	Denitrifying bioreactor	[Fe(II)EDTA]^2−^	${\mathrm{NO}}_{3}^{-}$	N_2_	Mixotrophic	Kumaraswamy et al., 2006
	*Paracoccus* sp.	Lab-scale serum bottles	nZVI	${\mathrm{NO}}_{3}^{-}$	N_2_	Mixotrophic	Jiang et al., 2015
*β*-Proteobacteria	*Acidovorax* sp. strain BoFeN1	Lake littoral sediments	FeCl_2_·6H_2_O	${\mathrm{NO}}_{3}^{-}$	${\mathrm{NO}}_{2}^{-}$, N_2_O, N_2_	Mixotrophic	Liu et al., 2019b
	*Alcaligenes eutrophus*	Anoxic flask	ZVI	${\mathrm{NO}}_{3}^{-}$	N_2_	Mixotrophic	Zhang et al., 2019c
	*Aquabacterium parvum* B6	Upflow bioreactor	FeCl_2_·4H_2_O	${\mathrm{NO}}_{3}^{-}$	N_2_	Mixotrophic	Zhang et al., 2016b
	*Azospira*	Lab-scale UCT-A/MBR	FeSO_4_·7H_2_O	${\mathrm{NO}}_{3}^{-}$	N_2_	Mixotrophic	Zhang et al., 2020a
	*Dechloromonas* sp. strain UWNR4	River sediments	Fe(II)-EDTA	${\mathrm{NO}}_{3}^{-}$	N_2_	Mixotrophic	Anirban and Flynn, 2013
	*Pseudogulbenkiania* sp. strain 2002	Freshwater sediments	FeSO_4_·7H_2_O	${\mathrm{NO}}_{3}^{-}$	N_2_O, N_2_	Mixotrophic	Chen et al., 2020a
	*Rhodopseudom onas*	CWs	Iron scraps	${\mathrm{NO}}_{3}^{-}$	N_2_	Mixotrophic	Ma et al., 2020
	*Thiobacillus* sp.	Black odorous sediment	Fe(II)	${\mathrm{NO}}_{3}^{-}$	${\mathrm{NO}}_{2}^{-}$	Autotrophic	Mai et al., 2021
	*Zoogloea* sp. L2	River sediments	Fe–C powder	${\mathrm{NO}}_{3}^{-}$	N_2_	Mixotrophic	Xu et al., 2021
*γ*-Proteobacteria	*Citrobacter freundii* strain PXL1	Anoxic activated sludge	FeCl_2_·4H_2_O	${\mathrm{NO}}_{3}^{-}$	N_2_	Mixotrophic	Li et al., 2015
	*Klebsiella mobilis*	Lab stock	FeSO_4_·7H_2_O,	${\mathrm{NO}}_{3}^{-}$	${\mathrm{NO}}_{2}^{-}$	Mixotrophic	Etique et al., 2014
	*Pseudomonas stutzeri* LS-2	Paddy soil	FeCl_2_·4H_2_O	${\mathrm{NO}}_{3}^{-}/{\mathrm{NO}}_{2}^{-}$	N_2_O, N_2_	Mixotrophic	Li et al., 2018a
	*Thermomonas* sp.	River surface sediments	Fe(II)	${\mathrm{NO}}_{3}^{-}/{\mathrm{NO}}_{2}^{-}$	N_2_	Mixotrophic	He et al., 2017
*δ*-Proteobacteria	*Desulfovibrio* sp. CMX	Lab-scale anaerobic vial	ZVI	${\mathrm{NO}}_{3}^{-}/{\mathrm{NO}}_{2}^{-}$	${\mathrm{NH}}_{4}^{+}$	Mixotrophic	Su et al., 2020

Nitrate/nitrite-dependent IOM can be divided into autotrophic and mixotrophic microorganisms in terms of metabolic types. Autotrophic IOM do not require organic carbon and use only Fe(II) as an electron donor to generate energy and fix carbon dioxide. However, such bacteria are rarely reported or distributed in wild environments. In contrast, mixotrophic IOM require organic carbon as a common reducing substrate with Fe(II). At present, it has been observed that 90% of nitrate-reducing bacteria can oxidize Fe(II) in the presence of organic matter. In other words, many heterotrophic denitrifying bacteria are also IOM in nature and are mostly heterotrophic or mixotrophic. These facts indicate great possibilities for the application of NDAFO with abundant and readily available functional microorganisms sources.

#### Electron Transfer Mechanism for NDAFO

**Figure 1:**
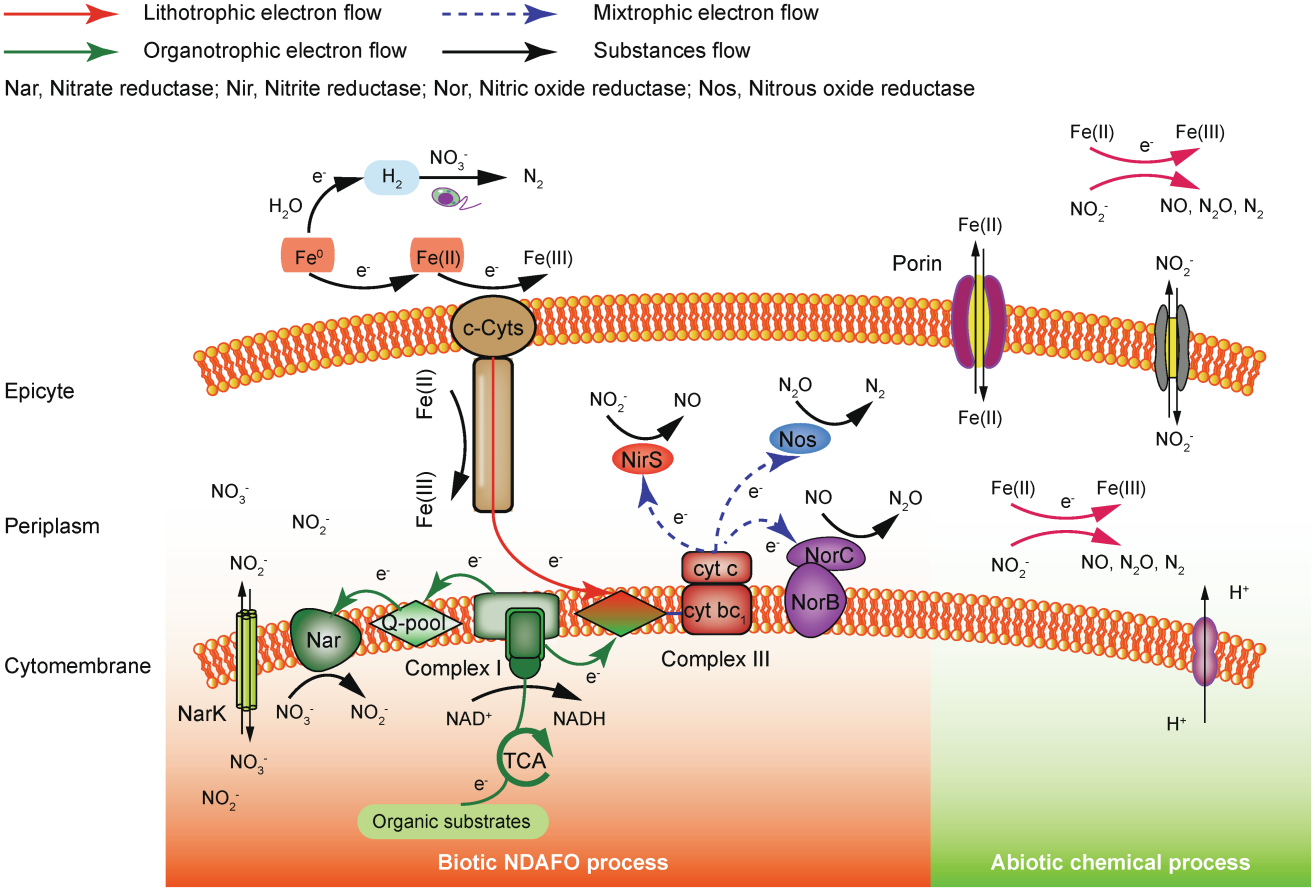
Electron transfer mechanism of the ZVI/Fe(II)-supported NDAFO process (Zhang et al., 2020b).

Currently, the precise electron transfer mechanism of enzyme-catalyzed NDAFO is not well understood. According to existing studies, electrons supplied by Fe(II) are transferred to ${\mathrm{NO}}_{3}^{-}$ and intermediate nitrogen species during denitrification by iron oxidoreductase, the quinone pool, NAR, cytochrome bc_1_ complex, etc., coupled with the conventional heterotrophic electron transfer chain (Figure 1). Although no specific enzyme has been identified to accept an electron from extracellular Fe(II), it has been reported that cytochrome *c* (*c*-Cyts) is likely involved in the electron transfer between Fe(II) and IOM; however, more direct evidence is needed for verification, and other enzyme proteins need to be evaluated (Liu et al., 2019a). In addition, Fe(II) can penetrate the outer membrane into the periplasm through porin-containing proteases and chemically react with ${\mathrm{NO}}_{2}^{-}$ to produce gaseous NO, N_2_O and N_2_.

### Microbial-Mediated Fe(III) Reduction

#### IRM for Feammox

Feammox plays an important role in nitrogen cycling globally, especially in Fe(III)-rich soils or wetlands. IRM are the dominant microorganisms in the Feammox process, which oxidizes ${\mathrm{NH}}_{4}^{+}$ to ${\mathrm{NO}}_{2}^{-}$, ${\mathrm{NO}}_{3}^{-}$ and N_2_ to produce bioenergy for cell growth (Eq. 20–22; Tan et al., 2021). In 1993, Lovley et al. (1993) first reported that *Geobacter metallireducens* could use Fe(III) as the sole electron acceptor to oxidize short-chain fatty acids, alcohols and monoaromatic compounds, which proved the existence of IRM. In 2005, Clément et al. (2005) accidentally detected the production of ${\mathrm{NO}}_{2}^{-}$ and Fe(II) in riparian wetland soils for the first time and observed dissimilar iron reduction by ammonium oxidation under anaerobic conditions. In 2006, Sawayama (2006) observed that ${\mathrm{NH}}_{4}^{+}$ was oxidized to ${\mathrm{NO}}_{2}^{-}$ in an anaerobic fixed-bed reactor supplemented with Fe(III)-EDTA and named the process “Feammox” (ferric ammonium oxidation). *Exiguobacterium* sp. was found to be the dominant IRM from high-throughput sequencing targeting the 16S rRNA gene. Subsequently, many different IRM have been observed to participate in the Feammox process, including Proteobacteria (Benaiges-Fernandez et al., 2019; Li et al., 2019; Ding et al., 2020a,b; Wang et al., 2021; Yang et al., 2021a), Actinobacteria (Shuai and Jaffé, 2019; Ma et al., 2021) and Firmicutes (Sawayama, 2006; Qin et al., 2019; Yang et al., 2019; Yao et al., 2020; Table 3). Among them, *Geobacteraceae* sp. and *Shewanella* sp. have been widely reported and studied. Although 16S rRNA high-through sequencing is promising to identify more IRM in recent studies, pure strain isolation in enrichment cultures is still needed to explore the mechanisms underlying Feammox.

**Table 3:** Fe(III)-reducing microorganisms for ${\mathrm{NH}}_{4}^{+}$ oxidation in Feammox.

Phylum	Species	Sample sites	Electron donors	Electron acceptors	Products	References
*β*-Proteobacteria	*Thiobacillus* sp.	Farmland soils	${\mathrm{NH}}_{4}^{+}$	Fe(III)	N_2_	Ding et al., 2020a
*γ*-Proteobacteria	*Pseudomonas* sp.	Paddy soil	${\mathrm{NH}}_{4}^{+}$	Fe(III)	N_2_	Li et al., 2019
	*Shewanella lohica*	Marine sediment	${\mathrm{NH}}_{4}^{+}$	Fe(III) oxides	N_2_	Benaiges-Fernandez et al., 2019
*δ*-Proteobacteria	*Anaeromyxobacter* sp.	Paddy soil	${\mathrm{NH}}_{4}^{+}$	Fe(III)	N_2_	Wang et al., 2021
	*Geobacteraceae*	Ecosystem habitats	${\mathrm{NH}}_{4}^{+}$	Fe(III)	${\mathrm{NO}}_{3}^{-}$, ${\mathrm{NO}}_{2}^{-}$, N_2_	Ding et al., 2020b
	*Geobacter* sp.	Anaerobic bottles	${\mathrm{NH}}_{4}^{+}$	Fe(III) coagulants	N_2_	Yang et al., 2021a
Actinobacteria	*Acidimicrobiaceae* sp. A6	CWs	${\mathrm{NH}}_{4}^{+}$	2-Line ferrihydrite	${\mathrm{NO}}_{2}^{-}$	Shuai and Jaffé, 2019
	*Geothrix* sp.	CWs	${\mathrm{NH}}_{4}^{+}$	Oxidized iron scraps	${\mathrm{NO}}_{2}^{-}$	Ma et al., 2021
Firmicutes	*Bacillus* sp.	Wheat-rice rotation area	${\mathrm{NH}}_{4}^{+}$	Fe(III)	N_2_	Qin et al., 2019
	*Desulfosporosinus* sp.	Anaerobic vials	${\mathrm{NH}}_{4}^{+}$	Fe_2_O_3_	N_2_	Yang et al., 2019
	*Exiguobacterium* sp.	Fixed-bed reactor	${\mathrm{NH}}_{4}^{+}$	Fe(III)-EDTA	N_2_	Sawayama, 2006
	*Fervidicella* sp.	Biofilm reactor	${\mathrm{NH}}_{4}^{+}$	Oxidized sponge iron	${\mathrm{NO}}_{3}^{-}$, N_2_	Yao et al., 2020

#### Electron Transfer Mechanism for Feammox

Many IRM rely on a series of redox-sensitive proteins or multiple heme cytochromes to transfer extracellular electrons by direct contact with ion minerals, which form an extracellular electron transfer (EET) pathway that binds the cell’s internal respiratory chain to external solid Fe(III) minerals. *Shewanella* sp. and *Geobacter* sp. often use *c*-Cyts to transfer electrons, exploiting the many solvent-exposed hemes as electron transfer centers, as heme comprises iron atoms and porphyrin rings.

**Figure 2:**
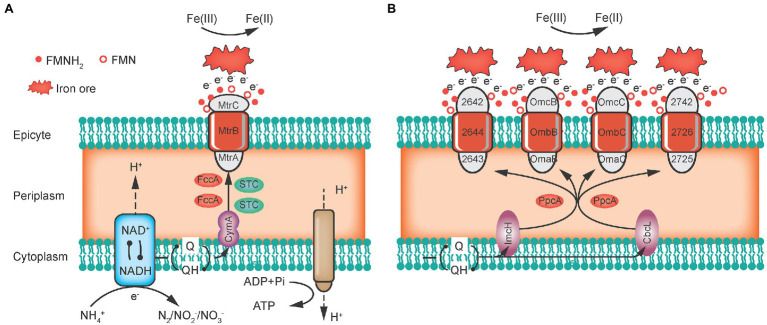
Extracellular electron transfer pathways of *Geobacter sulfurreducens* **(A)** and *Shewanella oneidensis* (**B**; Liu et al., 2018).

*Shewanella oneidensis* has 39 genes that encode *c*-Cyts, which are considered to be electron transport mediators, including CymA in the cytoplasmic membrane, Fcc3 and STC in the periplasm, and MtrCBA in the outer membrane (Figure 2A). CymA is a dehydrogenase that oxidizes quinols to release electrons and then transfers electrons directly or indirectly *via* Fcc3 and STC to MtrA. For the MtrCBA structure, MtrA is inserted into the porin-like protein MtrB and then interconnects with MtrC in the outer membrane, which finally forms a ternary trans-outer membrane complex. In addition, *Shewanella* sp. can also secrete small molecules, such as flavin and quinones, as electron shuttles (ESs) to achieve long-distance EET (Shi et al., 2016).

Regarding *Geobacter* sp., *G. sulfurreducens* has 111 genes encoding these *c*-Cyts, which can be divided into three categories (Figure 2B): (1) ImcH and CbcL first oxidize quinols to release electrons in the cytoplasmic membrane; (2) PpcA and PpcD receive and transfer electrons in the periplasm; and (3) Omas (B and C) and Omcs (B, C or Z) form trans-outer membrane protein complexes, which combine with porin-like proteins OmbB and OmbC to eventually transfer electrons to the extracellular space (Liu et al., 2018). Overall, electrons are derived from quinols in the cell’s inner membrane through periplasm transport and outer membrane emission to the final electron acceptor outside the cell.

### The Integration of Fe Oxidation and Reduction

#### The Coupling of Biotic and Abiotic Reactions

The reduction of ${\mathrm{NO}}_{3}^{-}$ to ${\mathrm{NO}}_{2}^{-}$ is the first step of microbial denitrification, which is catalyzed by membrane-bound nitrate reductase (NAR) or nitrate reductase in the periplasm (NAP). Depending on environmental conditions, such as the temperature, concentration of organics, pH, and aeration time, ${\mathrm{NO}}_{2}^{-}$ can accumulate to mM concentrations in the partial nitrification process. Microbially produced ${\mathrm{NO}}_{2}^{-}$ can be reduced by Fe(II) to gaseous NO, N_2_O, and N_2_ *via* chemodenitrification. In anoxic iron-containing environments, this process results from integrating biotic and abiotic processes, which jointly promote total nitrogen (TN) removal in the aqueous phase. This coupling provides a solution to the problem where Fe(II) does not react with ${\mathrm{NO}}_{3}^{-}$ in certain environments. However, accurate contribution ratios for the biotic and abiotic pathways should be quantitatively determined for an in-depth understanding of the denitrification process.

#### The Coupling of Microbial Fe(II) Oxidation and Fe(III) Reduction

**Figure 3:**
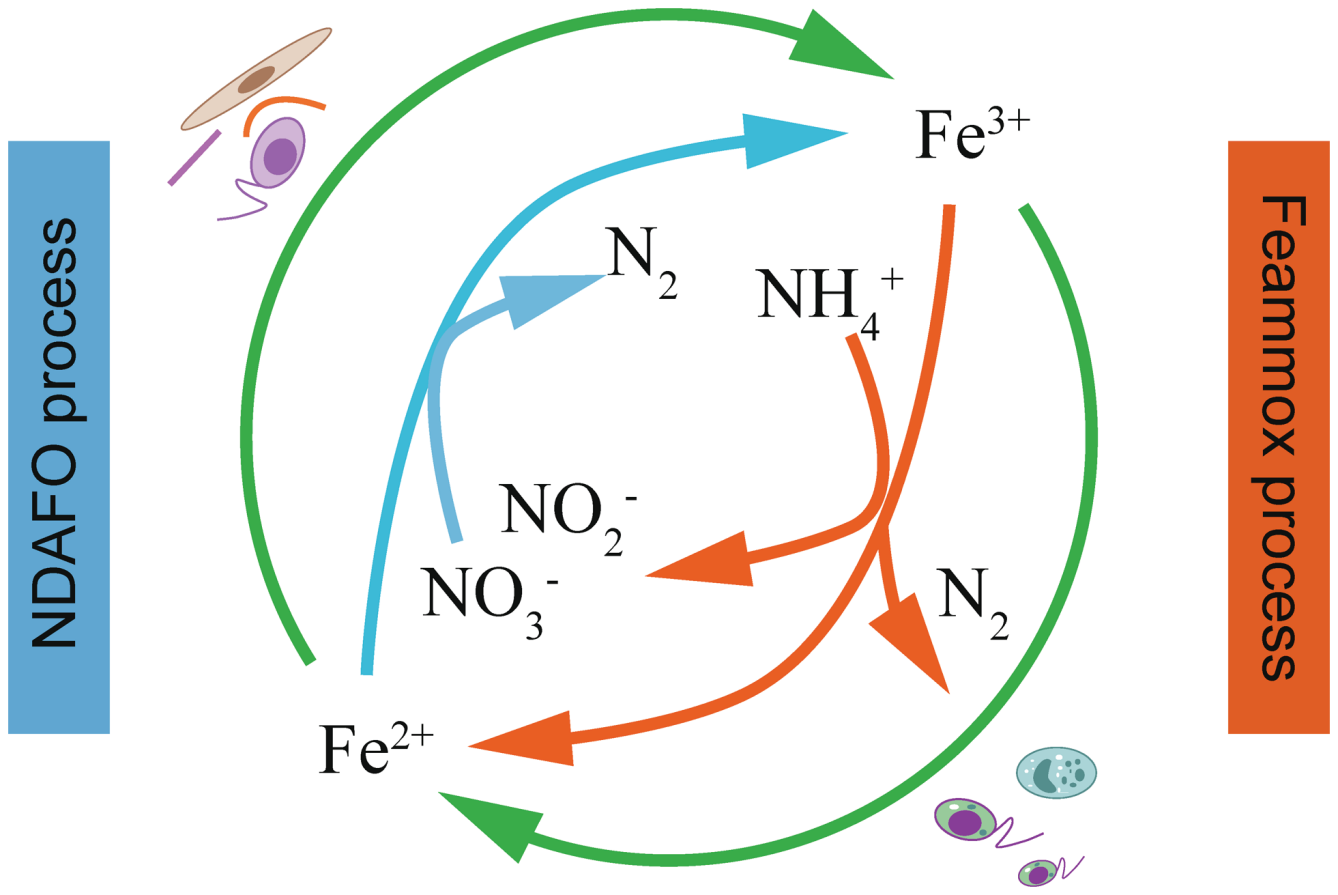
The coupling of the Feammox and NDAFO processes for TN removal (Li et al., 2018b).

Some Fe(III) minerals, as electron acceptors, could biotically oxidize ${\mathrm{NH}}_{4}^{+}$ in IRM-mediated Fe(III) reduction, and the Fe(II; hydr)oxides produced in the above process might be utilized as electron donors and react with ${\mathrm{NO}}_{x}^{-}$ in anaerobic conditions (Figure 3). The biotic enzymatic Fe(III) reduction and Fe(II) oxidation rates are generally higher than those of abiotic chemical reactions in nitrogen-rich conditions (Lovley, 1997). This integration has important implications for microbial ecology since specific bacteria can gain chemical energy for growth from the two energy generation processes. Although the IRM and IOM might be spatially or temporally separated, a syntrophic relationship is formed in which iron-bearing minerals mutually support the growth of each group (Zhao et al., 2015). Yang et al. (2020) developed an Anammox-like process to treat sludge digest effluent with high-concentration ${\mathrm{NH}}_{4}^{+}$ using ${\mathrm{NO}}_{3}^{-}$ as a terminal electron acceptor in the Fe(III)/Fe(II) cycle, in which ${\mathrm{NH}}_{4}^{+}$ was mainly oxidized by Fe(III) to N_2_ through Feammox. The produced Fe(II) triggered NDAFO to reduce ${\mathrm{NO}}_{x}^{-}$ to achieve effective TN removal. The coupled processes provide a safe and efficient method to treat ${\mathrm{NH}}_{4}^{+}$ and ${\mathrm{NO}}_{3}^{-}$ wastewater simultaneously.

## Environmental Factors

### pH

The environmental pH generally has an important effect on iron speciation. At extremely low pH, Fe(II) is oxidized to Fe(III), which exists in the form of free ions or iron complexes under aerobic conditions. When the pH is over 4, aqueous Fe(II) is easily transformed to structural Fe(III) that precipitates as poorly crystalline iron minerals (Johnson et al., 2012). For example, at low pH, reduced nontronite containing structural Fe(II) can be oxidized to aqueous Fe(III), but the high pH is not favorable for this electron transfer process due to the precipitation of aqueous Fe(III; Guo et al., 2020). Thus, pH has a significant impact on the form and availability of iron.

Furthermore, microbial iron-associated metabolism also has significant implications for controlling the environmental pH of the microbial habit. For example, pH is the critical control parameter for maintaining sustainable fermentation, but pH could constantly decrease as organic substrates *(*e.g., glucose or pyruvate) are consumed. Fermentative iron reducers, such as *Orenia metallireducens* strain Z6, enhanced the degradation of fermentable substrates in the presence of hematite and effectively generated alkalinity that balances acid production, providing favorable buffering conditions for microbial fermentation (Dong et al., 2017). Similarly, ZVI can consume protons in the solution, thus increasing the pH in the reaction system (Eq. 7). In summary, the presence of iron minerals can affect environmental pH indirectly or directly.

### Oxidation–Reduction Potential

**Figure 4:**
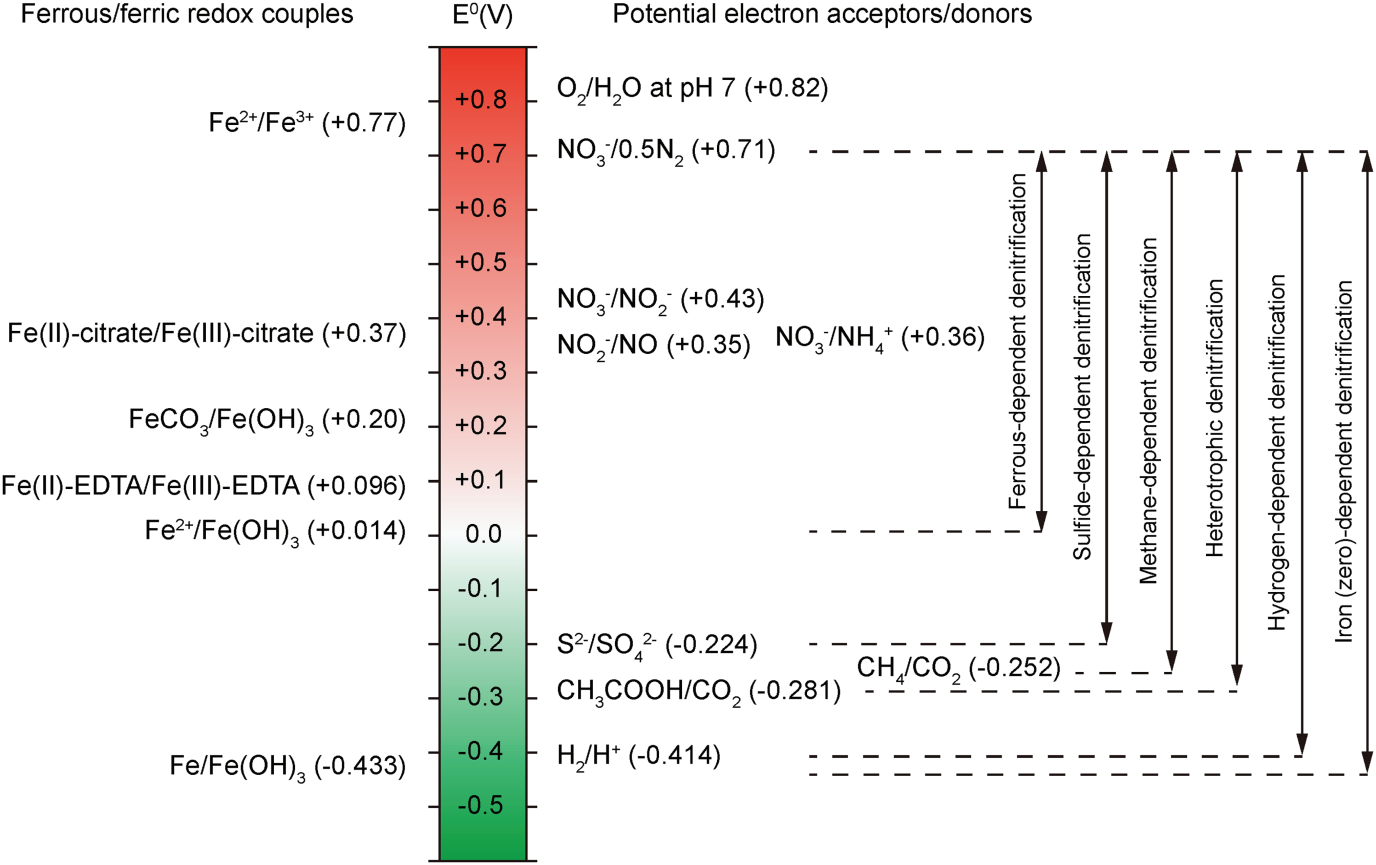
Redox potential of Fe and N species couples in NDAFO; the double-headed arrows refer to the ΔORP between different denitrification processes (Hedrich et al., 2011).

ORP is another crucial parameter that affects the removal of nitrogen in an anaerobic BNR system. As shown in Figure 4, at neutral pH, the ORP of Fe(II)/Fe(OH)_3_ is +0.014 V, which is lower than that for any denitrifying nitrogen intermediate couple of ${\mathrm{NO}}_{3}^{-}$/0.5N_2_ (+0.71 V), ${\mathrm{NO}}_{3}^{-}/{\mathrm{NO}}_{2}^{-}$ (+0.43 V) and NO_3_^−^/NO (+0.35 V). Different ${\mathrm{NO}}_{3}^{-}$ reduction products are associated with different ORPs, resulting in different bioenergies obtained by IOM. In addition, microbes get different energy from different types of denitrification processes due to the ORP difference (ΔORP). Note that the ΔORP of ferrous-dependent denitrification is smaller than that of sulfide-, methane-, heterotrophic- and hydrogen-dependent denitrification, which means NDAFO microorganisms may derive less energy from a sole Fe(II) oxidation process. It is reported that a few IOM strains were found to survive under autotrophic conditions just using Fe(II) as electron donor, and most NDAFO microorganisms are mixotrophic to simultaneously obtain energy from organic matter (Liu et al., 2019a). Furthermore, ZVI-dependent denitrification has a larger ΔORP than the other denitrification pathways, providing an efficient potential for ${\mathrm{NO}}_{3}^{-}$ reduction.

Some anaerobic microbes can create low-redox-potential conditions for growth and preservation by reducing a range of oxidized compounds into products with a rather low reduction potential. *Methanosarcina barkeri* utilizes hydrogen gas (H_2_) as an electron donor and ferredoxin (E^0^’ = −500 mV) and coenzyme F_420_ (E^0^’ = −360 mV) as electron carriers to reduce Fe(III) to Fe(II), such as FeOOH(am) → Fe(II), E^0^’ = −50 mV, or ZVI, such as Fe(III) + 3*e^−^* → Fe(0), E^0^’ = −37 mV (Shang et al., 2020). Additionally, the increase in Fe(II) concentration can remarkably decrease the redox potential and favor denitrification by microorganisms. A high TN removal efficiency was observed with external addition of 50 mg/l Fe(II) in a horizontal subsurface flow CW (Zhang et al., 2019d). Thus, the relatively low ORP environment created by the microorganism itself or artificial measures contributes to the Feammox or NDAFO process.

### Fe Species

Fe mineralogical properties such as crystallinity have an important role in IOM and IRM bioavailability, affecting the rate and extent of Fe reduction and oxidation. The metabolic rate is generally inversely proportional to mineral crystallinity. For example, Fe(II) in clay minerals contains structural sites, Fe(II)-complexed surface hydroxyl group (edge) sites and basal/interlayer sites, and only structural and edge Fe(II) sites are highly reactive toward ${\mathrm{NO}}_{3}^{-}$ reduction (Zhang et al., 2019b). Low-crystallinity Fe(III; hydr)oxides, such as ferrihydrite, for microbial Fe(III) reduction were found earlier than crystalline Fe(III) minerals, such as goethite, lepidocrocite and magnetite. In microbial Fe(III) reduction, although the mineral type is the same, goethite with more defects could produce more reduced atoms (ferrous iron ions) than goethite with fewer defects (Notini et al., 2019).

Moreover, microbial metabolism and environmental conditions can also bring about the evolution of Fe species. Mejia et al. (2016) compared the transformation of lepidocrocite and ferrihydrite with soil microbial communities at the interface of anaerobic/anoxic environments during redox cycling, and magnetite was observed as the same main product of both mineral reductions. Additionally, subsequent Fe(II) oxidation by O_2_ promoted the production of ferrihydrite and lepidocrocite, which are accessible to IRM for redox cycling but formed nonstoichiometric and low-bioavailability magnetite during the ${\mathrm{NO}}_{3}^{-}$ reduction periods.

#### Fe(II)

In the Fe(II)-driven denitrification system, biogenic Fe(III; hydr)oxides largely depend on different inoculated denitrifiers. A *Thiobacillus*-dominated mixed culture converted poorly crystalline ferrihydrite to crystalline akaganeite, while *T. denitrificans* and *Pseudogulbenkiania* strain 2002 preferentially produced maghemite and hematite (Kiskira et al., 2019). This in-depth understanding of microbial factors is critical to predicting the fate and recovery of Fe species in natural or engineered conditions.

#### Fe(III)

Ferrihydrite is a thermodynamically metastable iron (hydr)oxide that can be gradually transformed to crystalline goethite and hematite. (Bi)carbonate levels that are elevated (pCO_2_, ~2%) compared to atmospheric CO_2_ levels (~0.04%), a critical geochemical parameter in sediments, could increase the occurrence of hematite through olation, ligand exchange, and rearrangement (Li et al., 2020b). Under suboxic conditions, aqueous Fe(II) can catalyze ferrihydrite transformation to more stable lepidocrocite (Lp) and goethite (Gt), and citrate greatly affects the ratio of Lp to Gt (Sheng et al., 2020a).

### Extracellular Electron Transfer

**Figure 5:**
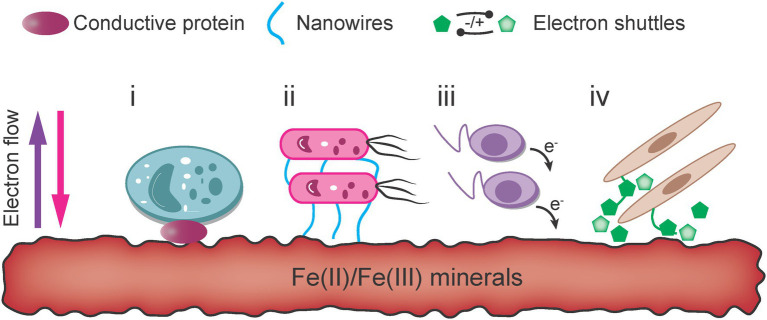
Mechanisms of extracellular electron transfer for iron-metabolizing extracellular respiratory bacteria (Li et al., 2021a).

The primary sources of Fe(III)/Fe(II) oxides that microorganisms can access are highly insoluble in natural environments. Thus, the dissolution of solid iron phases becomes a key process affecting the bioavailability of iron minerals. Through extracellular respiration, bacteria directly contact or link with iron oxides through specific physiological nanowires to transfer electrons, generally at a low rate that is highly dependent on the species. Indirect electron transfer between microbes and ore can be achieved with ligands, ESs and other microbial Fe acquisition strategies (Figure 5).

#### Fe(II)

Ligand and ESs have been reported to facilitate electron transfer between Fe(II) and IOM, enhancing microbial Fe(II) oxidation in wastewater treatment. *c*-Cyts is a key protein involved in IOM extracellular electron transfer. Organic ligands were detected with a rapid stopped-flow spectrometer and found to significantly accelerate the reaction between *c*-Cyts and Fe(II), with a reaction rate order of EDTA > citric acid > ammonia triacetate > malonic acid > glycine amino acid > control (Wang et al., 2019b). In addition, Fe(II) can be oxidized by ESs, e.g., oxidized riboflavin, flavin mononucleotide (FMN) and quinone, providing bioreinforcement strategies for the reduction of redox-sensitive nitrogen species (Zhang et al., 2020c). Thus, ESs are demonstrated a feasible and efficient strategy to promote NDAFO process.

#### Fe(III)

The addition of iron ligands, such as nitrilotriacetic acid (NTA), ethylenediaminetetraacetic acid (EDTA), *N*-methyliminodiacetic acid (MIDA) and polyphosphate significantly accelerated Fe(III) reduction coupled to ammonium oxidation. Synergetic effects of submicromolar Fe(II) addition and ligands catalyzed the dissolution of lepidocrocite due to interfacial electron transfer of Fe(III)/Fe(II) and detachment of Fe ligands (Biswakarma et al., 2020). The extent of Feammox and the reduction of ferrihydrite were enhanced when amended with the ES AQDS (Zhou et al., 2016). Biochar facilitated long-term microbial reduction of hematite at a two fold higher rate than that in the control since the semiquinone groups on biochar likely participated in the redox reactions (Xu et al., 2016). Hence, ES-mediated Feammox could lead to greater N loss and a promoted reaction rate in wastewater.

### Natural Organic Matter

#### ZVI/Fe(II)

NOM is abundant in aquatic environments and has a critical impact on FeBNR. In a ZVI-oxidizing supported autotrophic denitrification process based on iron-carbon microelectrolysis and iron scraps (ME-ISs-AD), an optimum dosage of 1.0 mg-COD/mg-TN can significantly increase the denitrification load from 0.19 to 0.44 kg-N/(m^3^·d) and reduce the accumulation of N_2_O/NO_2_^−^. The study demonstrated that organic carbon simulated the bioconversion of iron compounds and enhanced ZVI passivation, increasing the production of H_2_ and Fe(II) and promoting autotrophic denitrification (Deng et al., 2020). NOM has positive and negative influences on the NDAFO process. On the one hand, NOM can behave as an electron donor to enrich mixotrophic IOM and increase bacterial activity. On the other hand, Fe(II)-NOM complexes cannot enter the periplasm to participate in electron transport chains. Aqueous Fe(II) may react enzymatically or abiotically with ${\mathrm{NO}}_{2}^{-}$ in the periplasm or at the surface of cells. The inhibition of denitrification by Fe(II)-OM was observed due to the large size and negative charge of such complexes (Peng et al., 2018).

#### Fe(III)

Under anaerobic conditions with ferric ions as the sole electron acceptor, NOM can compete with ${\mathrm{NH}}_{4}^{+}$ as the electron donor for Feammox. Therefore, ${\mathrm{NH}}_{4}^{+}$ is preferentially utilized in the absence of organic carbon. In wild environments, such as saline-alkaline paddy soils, ${\mathrm{NH}}_{4}^{+}$ is generally uncorrelated or negatively related to NOM under Fe(III)-dominant reducing conditions (Liu et al., 2021). However, recent studies showed that NOM was favorable for Feammox because (1) organic carbon can promote the release of structural Fe in clay minerals, supporting the release of amorphous Fe and promoting the bioavailability of Fe minerals (Glodowska et al., 2020); (2) some organic carbon, such as a humic substances, can act as electron donors and ESs to link insoluble Fe(III) and IRM (Stern et al., 2018); and (3) the degradation of organic carbon can release protons, which leads to a decrease in pH and directly provides H^+^ to the Feammox process. Although organic carbon is not required for Feammox metabolism, it does play a key role in mediating and enhancing Feammox rates.

## Processes and Applications

### Natural Water Systems and Wetlands

#### Groundwater

Nitrogen pollution in groundwater is a pervasive and increasing global issue mainly due to the intense application of nitrogen fertilizers, affecting human health if exposure is long-term. Nitrate is usually reduced to N_2_ by denitrifying bacteria and removed from drinking water, but this process consumes extensive amounts of organic carbon and possibly causes secondary pollution. Techniques utilizing Fe minerals provide alternative cost-effective solutions to remediate nitrogen-polluted groundwater.

##### ZVI/Fe(II)

The nanosized Fe(II)-containing mineral magnetite was proven to reduce ${\mathrm{NO}}_{3}^{-}$ to N_2_ in a batch environment with groundwater and sediments derived from wells located in Barcelona, Spain, to simulate aquifer conditions (Jiang et al., 2019). A similar study reported that magnetite nanoparticles could complete ${\mathrm{NO}}_{3}^{-}$ reduction to ${\mathrm{NO}}_{2}^{-}$ coupled to biological Fe(II) oxidation, and dissolved Fe(II) mainly contributed to abiotic ${\mathrm{NO}}_{2}^{-}$ reduction (Margalef-Marti et al., 2020). Li et al. (2015) adopted a single anaerobic nitrate-reducing Fe(II)-oxidizing *Citrobacter freundii* strain PXL1 for the simultaneous removal of arsenic and ${\mathrm{NO}}_{3}^{-}$ from groundwater. As an economical and available material, ZVI is widely used in remediating ${\mathrm{NO}}_{3}^{-}$-contaminated groundwater, but iron passivation has always been a complex problem to be solved effectively. More long-term methods to prevent passion or keep the activity of ZVI are the subject of future research.

##### Fe(III)

**Figure 6:**
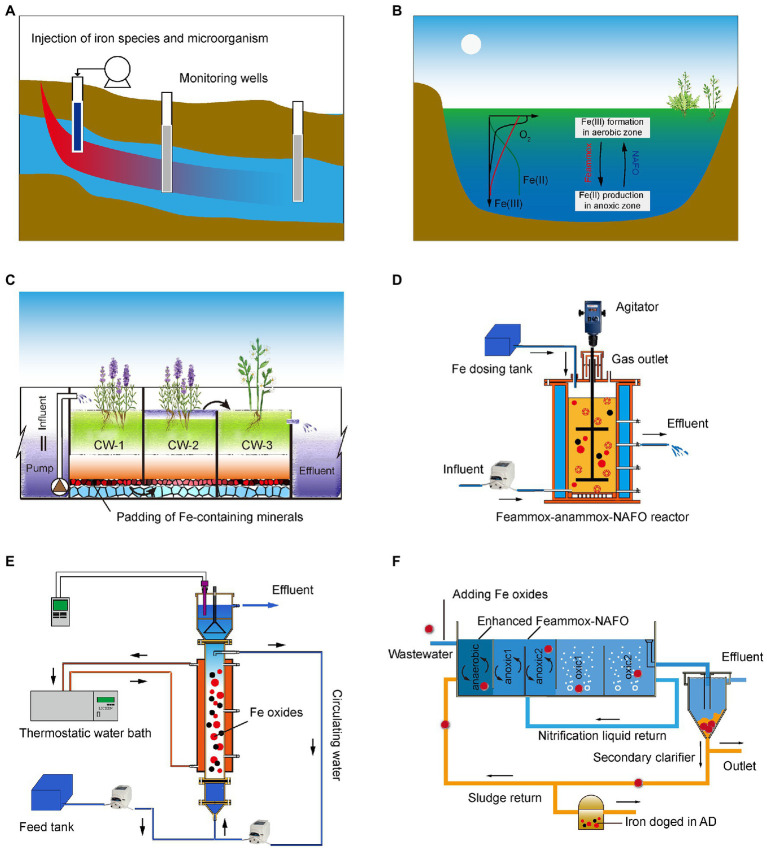
Application scenarios for FeBNR in wastewater treatment processes for groundwater **(A)**; lake and river water **(B)**; CWs **(C)**; sequencing biological reactor **(D)**; continuous-flow reactor **(E)** and mainstream waste water treatment plants (WWTPs) process (**F**; Zhao et al., 2016).

Fe(III; hydr)oxides, functional IRM and ESs could be injected into groundwater aquifers to remove ${\mathrm{NH}}_{4}^{+}$ by constructing injection wells, permeable reactive barriers (PRBs) or Kariz systems (also known as canals), as shown in Figure 6 (Jiang et al., 2019). Spearman analysis between the geochemical parameters and microbial community in the western Hetao Basin of China indicated a widespread occurrence of Feammox in groundwater systems (Xiu et al., 2020).

#### Surface Water Ecosystems

##### ZVI/Fe(II)

Nitrogen biotransformation regulated by FeBNR has been observed in ecosystem habitats, rivers and natural wetlands. Urbanization has increased the nitrogen pollution in sediments of urban river networks, and Pearson correlation analysis showed that Fe(II) had a significant influence on the ${\mathrm{NO}}_{3}^{-}$ reduction process, contributing to TN loss in an investigated river network in Shanghai, China (Zhang et al., 2019d). In the freshwater lake Almind (Silkeborg, Denmark), the addition of Fe(II) simulated nitrate reduction to N_2_ (Robertson and Thamdrup, 2017). The occurrences of NDAFO provide a potential nitrogen mitigation pathway to purify surface water.

##### Fe(III)

Agricultural fertilizer is a great producer and emitter of nitrogen into surrounding water bodies, and Feammox in agricultural drainage ditches was reported to mitigate nitrogen pollution in the Jiuli River, Taihu Lake Basin (Chen et al., 2020b). A further field investigation in these watersheds in China indicated that the potential Feammox rates varied from 2.4 to 22.5 kg-N/(ha·yr) both seasonally and spatially within the investigated farmland, riparian land and sediment (Ding et al., 2020b). An ^15^N-labeled isotopic tracing technique indicated that a loss of 12.33 t-N/y was associated with Feammox, accounting for 6.4–6.7% of TN loss through N_2_, in the Jiulong River estuary, China (Guan et al., 2018). Thus, more reports demonstrated that Feammox is a vital biochemical nitrogen cycle in aquatic ecosystems.

#### Constructed Wetlands

A CW could be designed and built on an *in situ* polluted site to control nitrogen transformation, decrease transportation fees, and avoid excessive interference with the local environment. However, nitrogen removal is always limited by the absence of electron donors in CWs. Autotrophic denitrification, especially with iron supplements, has become a practical alternative method to enhance nitrogen removal. The complicated interactions between environmental parameters, substrates, and microorganisms significantly affect nitrogen transformation in iron-based CWs and have been the focus of many studies.

##### ZVI/Fe(II)

Regarding iron as an electron donor, iron-containing materials, such as iron scraps, iron ore and steel slag, can be chosen as supporting substrates for autotrophic denitrification. The coupling in ferric-carbon microelectrolysis enhanced the TN removal efficiency to 90.5% from 31.4% in ordinary subsurface-flow CWs (Shen et al., 2019). The production of H_2_/[H] on the cathode and Fe(II) from ZVI on the anode facilitated the enrichment of facultative autotrophic denitrifiers, leading to further ${\mathrm{NO}}_{3}^{-}$ reduction. For example, with nZVI as a cosubstrate, modified agricultural wastes as solid carbon sources (SCSs) further enhanced the ${\mathrm{NO}}_{3}^{-}$ removal efficiency (75.3–91.9%) compared with that in SCSs-CW alone (63.3–65.5%; Zhao et al., 2019). In addition, microelectrolysis transforms macromolecular organic matter into micromolecular organic matter, which is a suitable biodegraded carbon source for biological denitrification. Various Fe(II)-containing minerals, such as siderite, pyrite, pyrrhotite, and biotite, have been added to CWs to enhance denitrification.

##### Fe(III)

Fe(III)-containing materials, such as ferrihydrite, goethite and lepidocrocite, have been applied to anaerobic ammonium oxidation. Feammox was enhanced in a CW by incubating with functional *Acidimicrobiaceae* sp. A6 and increasing the content of ferrihydrite (Shuai and Jaffé, 2019). Yang et al. (2021b) developed an integrated CW-microbial fuel cell system (CW-MFCs) with pyrrhotite as one of the supported substances, and Feammox and pyrrhotite-based autotrophic denitrification co-occurred, increasing the ${\mathrm{NH}}_{4}^{+}$ removal efficiency by 87%. The addition of iron minerals can improve the contribution of autotrophic nitrogen removal in CW, and further research should focus on new composites and operating conditions for applications.

### Artificial Water Treatment System

#### Sequencing Batch Reactor

##### ZVI/Fe(II)

The SBR is characterized by simplicity, low cost and flexibility of operation, which are good advantages for cultivating slow-growing bacteria enriched in a reactor. In an SBR treating digested effluent, the addition of ZVI provided electrons for denitrification, and the produced Fe(II) was favorable for Anammox enrichment (Wang et al., 2018). A nZVI–supported denitrifying bacteria, *Alcaligenes eutrophus*, was cultivated in a cylindrical culture tank, and the results of this study demonstrated that ferrous iron [Fe(II)(ad)] adsorbed on ferric oxides is likely an effective electron donor for ${\mathrm{NO}}_{3}^{-}$ removal (Xu et al., 2018).

##### Fe(III)

Feng et al. (2020) chose SBR to enrich both marine Anammox bacteria (MAB) and autotrophic Feammox bacteria to treat nitrogen-laden saline wastewater with Fe(III) addition, achieving a maximal substrate conversion rate of 2.97–3.47 kg/(m^3^·d). Even under low-temperature and high-salinity conditions, Fe(III) addition still greatly improved the ${\mathrm{NH}}_{4}^{+}$ removal rate and had no adverse effect on MAB activity (Li et al., 2020a). Simultaneous nitrification and denitrification coupled with Fe redox cycling were achieved in SBR, treating domestic sewage and integrating the internal recycling of nanostructured Fe oxyhydroxides (FeOOH). The ${\mathrm{NH}}_{4}^{+}$ removal reached 73.1 ± 17.4 mg/l/d when associated with biogenic Fe(III) reduction (Desireddy et al., 2020). According to the stoichiometric amount of Fe(III) in the Feammox process, it is required to continuously or intermittently supply Fe(III) to maintain a continuous nitrogen removal but obviously uneconomical in practical application. The intermittent addition of ${\mathrm{NO}}_{3}^{-}$ or aeration might be an appropriate strategy to oxidize Fe(II) to produce secondary Fe(III) for the next round of Feammox.

#### Continuous-Flow Reactor

##### ZVI

ZVI-supported autotrophic denitrification has been developed for decades due to the well-documented characteristics of ZVI-based materials. Various continuous-flow processes are conducted for nitrate-contaminated wastewater treatment, including iron-(activated carbon) microelectrolysis-based carrier (IA-MEC) reactors and anoxic fluidized-bed membrane bioreactors (AnFB-MBRs; Peng et al., 2020). Generally, the products of nitrate reduction by ZVI are ${\mathrm{NH}}_{4}^{+}$ as the main product and little ${\mathrm{NO}}_{2}^{-}$ (the intermediate product) and N_2_; then, the corresponding ZVI is converted to ferrous or ferric oxides. On the basis of these traits Khalil et al. (2018) designed a laboratory-scale continuous-flow system (LSCFS) using reactor-settler-polisher techniques. The reagents nZVI, bimetallic nZVI-Cu and CuCl_2_-added nZVI were dosed into the reactor to transform ${\mathrm{NO}}_{3}^{-}$ with a nitrogen removal efficiency above 90%. A coupling of Anammox and ZVI/Fe(II) reduction was achieved in a continuous-flow expanded granular sludge blanket (EGSB) to enhance autotrophic nitrogen removal, in which iron-dependent dissimilatory nitrate reduction to nitrite or ammonia positively supported the Anammox process (Bi et al., 2019).

##### Fe(II)

Fe(II)-driven autotrophic denitrification has been achieved in upflow packed bed reactors (PBRs) and ferrous iron-based chemoautotrophic denitrification (Fe-CAD) reactors (Wang et al., 2017; Kiskira et al., 2020). NO has low solubility in aqueous solution and is generally harder to degrade with conventional biological techniques, but Fe(II)-EDTA exhibits strong complexation and high adsorption on it. Hence, Zhang et al. (2019a) developed an autotrophic upflow bioreactor with a sponge iron bed to combine NO adsorption and Anammox process for greater nitrogen removal, and the NDAFO rate was also increased. The autotrophic sulfur-based denitrification process has been widely adopted for ${\mathrm{NO}}_{3}^{-}$ removal to avoid the external addition of organic carbon. However, the produced acid is unfavorable to microbial growth and necessitates continual adjustment of the pH by limestone, NaOH solution, etc. Zhu et al. (2019) proposed a sulfur coupled with iron(II) carbonate-driven autotrophic denitrification (SICAD) system, in which siderite (FeCO_3_) was leached from the ore and promoted the NDAFO process. Similar ${\mathrm{NO}}_{3}^{-}$ and phosphate removal rates were achieved in a sulfur-siderite autotrophic denitrification (SSAD) system showing the feasibility of the pilot experiments using columns and pilot biofilters (SSAD-PB; Wang et al., 2019a).

##### Fe(III)

In 2015, Huang and Jaffé (2015) operated a continuous-flow membrane Feammox reactor with added ferrihydrite and ${\mathrm{NH}}_{4}^{+}$. After 180 days of incubation, uncultured *Acidimicrobiaceae* bacterium A6 was enriched in this reactor. Despite the fact that Feammox has recently received considerable attention, large-scale applications for mainstream WWTPs have rarely been reported. Feammox technology can be combined with other BNR processes (Zhu et al., 2021). For example, in a conventional A^2^/O process, even though nitrification must occur in an aerobic tank, external ${\mathrm{NH}}_{4}^{+}$ can be oxidized in an anaerobic or anoxic tank to increase the ${\mathrm{NH}}_{4}^{+}$ removal efficiency. The products of ${\mathrm{NO}}_{2}^{-}$ can be coupled with Anammox in an anoxic environment to increase the contribution of autotrophic BNR. Feammox can be similarly applied in a sidestream AD system with a higher ${\mathrm{NH}}_{4}^{+}$ load.

## Perspectives on FeBNR

In the past 30 years, nitrogen control and remediation methods for eutrophic lakes have mainly included chemical methods (addition of CuSO_4_ or herbicides to control algae blooms), physical methods (dilution and flushing, deep aeration, sediment dredging) and biological methods (microbial remediation, aquatic phytoremediation). FeBNR provide alternative or enhanced methods to integrate with the above techniques. For example, (1) covering sediments with of iron minerals *in situ* could hinder the release of ${\mathrm{NH}}_{4}^{+}$ from contaminated bottom sediments and absorb and biodegrade the ${\mathrm{NH}}_{4}^{+}$ *via* Feammox process in these sediments; (2) a combination of aquatic ecological floating beds and FeBNR on the mat not only mitigates the seasonal impact on and the treatment efficiency due to plant growth period but also notably improves the water purification; (3) in field remediation, since the landform and water depth determine the species and fates of nitrogen in lakes (Qin et al., 2020), the selected Fe(II) or Fe(III)-containing substrates and their addition amount should be based on the corresponding conditions; (4) if the iron mineral source is rich in some local or nearby sites, water transfer projects might be investigated and designed to utilize these Fe minerals to reduce N pollution. For Fe-poor conditions, iron supplementation with an *in situ* covering technique could be considered for further N removal in shallow or deep lakes. Fe and N cycles are tightly coupled as important biogeochemical processes, enabling multiple remediation techniques for nitrogen control in aquatic and terrestrial systems. Although the integration of Feammox and NDAFO is an emerging method to reduce TN, further studies should focus on the following:

1. The accurate stoichiometry between the Fe and N loads. In treating wastewater with high concentrations of ${\mathrm{NH}}_{4}^{+}$ using Feammox, the produced Fe(II) is generally inadequately oxidized to Fe(III) due to the absence of oxidizers, i.e., ${\mathrm{NO}}_{3}^{-}$ or ${\mathrm{NO}}_{2}^{-}$, hindering the next round of Feammox. Thus, the moderate N loads and proportion should match the well-balanced Fe(III)/Fe(II);
2. The identification and bioavailability of secondary biogenic minerals. Fe speciation and properties have significant influences on the rate and extent of ore bioavailability, and the optimal selectivity of Fe species could be favourable to enrich iron oxidizers or reducers, and operational parameters affecting Fe species transformation should be optimized to benefit the nitrogen removal efficiency;
3. The distinction of nitrogen transformation pathways. The complex substances in actual wastewater determine the diversity of nitrogen loss pathways, and heterotrophic denitrification, Anammox, Feammox and NDAFO might coexist in the same environment. Quantitatively differentiating the contributions of each pathway could help to determine proper control strategies;
4. FeBNR provides an effective method for eliminating nitrogen contaminants, and the rate limitation of FeBNR could be relieved by ESs. Biochar, as ES, is easy to prepare and widely used in practical projects. Hence, optimizing the key parameters of biochar production and catalysis for the FeBNR process deserves further study. The redox properties of biochar could be improved by adjusting the hydrothermal temperature or surface modifications, etc.

## Conclusion

Autotrophic FeBNR, coupling NDAFO with the Feammox process, has excellent potential to mitigate total nitrogen pollution with low C/N wastewater. This paper systematically summarized the recent advances in FeBNR technologies, especially mechanisms, microorganisms, and environmental factors that affect its reactive rate in natural habitats and engineered systems. The nitrogen conversion rate is generally low due to multiple environmental factors, and ESs provides an emerging strategy to facilitate the iron cycle by accelerating extracellular electron transfer. Furthermore, nitrogen transformation pathways between abiotic and biotic reactions should be accurately distinguished, and the effects of secondary minerals on the FeBNR process might be investigated in future studies. The novel FeBNR process is conducive to reducing organic carbon sources and energy consumption for future sustainable wastewater treatment.

## Author Contributions

SP: project administration, funding acquisition, writing—review and editing, and supervision. NL: conceptualization, writing—original draft, and investigation. HL: validation and writing—review and editing. XL: writing—original draft and visualization preparation. TS: visualization preparation and data curation. YY: validation and data curation. JJ: investigation, resources, and data curation. All authors contributed to the article and approved the submitted version.

## Funding

This work was supported by the National Natural Science Foundation of China (42077160, 52000039, and 52100004) and Jilin Province Natural Science Funds (20200201041JC).

## Conflict of Interest

The authors declare that the research was conducted in the absence of any commercial or financial relationships that could be construed as a potential conflict of interest.

## Publisher’s Note

All claims expressed in this article are solely those of the authors and do not necessarily represent those of their affiliated organizations, or those of the publisher, the editors and the reviewers. Any product that may be evaluated in this article, or claim that may be made by its manufacturer, is not guaranteed or endorsed by the publisher.
